# Epstein–Barr Virus-Associated Atraumatic Splenic Rupture

**DOI:** 10.5334/jbsr.3536

**Published:** 2024-03-21

**Authors:** Eva De Vis, Lauren Keuleers, Steven Dymarkowksi

**Affiliations:** 1 University Hospitals Leuven, Herestraat 49, 3000 Leuven, Belgium; 2 University Hospitals Leuven, Herestraat 49, 3000 Leuven, Belgium; 3 University Hospitals Leuven, Herestraat 49, 3000 Leuven, Belgium

**Keywords:** infectious mononucleosis, CT, atraumatic splenic rupture

## Abstract

*Teaching point:* Atraumatic splenic rupture is a rare but life-threatening complication of infectious mononucleosis.

## Case History

A 24-year-old woman presented at the emergency department with a 3-day history of feeling unwell, followed by the acute onset of atraumatic, severe abdominal pain. A physical examination showed an uncomfortable patient with diffuse abdominal pain and bilateral cervical lymphadenopathies. She had chills without fever, tachycardia and normal blood pressure.

Laboratory investigation revealed a moderate inflammatory blood count, mildly elevated liver function tests and a reduced haemoglobin level. The presence of Epstein–Barr virus (EBV) antibodies was compatible with an acute primary infection.

**Figure 1:**
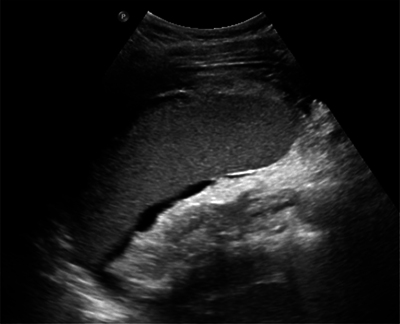
Abdominal ultrasound shows splenomegaly and a perisplenic, layered fluid collection.

Ultrasound in the emergency setting showed abdominal and pelvic free fluid, splenomegaly and a perisplenic, layered hyperechogenic fluid collection (Figure 1).

**Figure 2:**
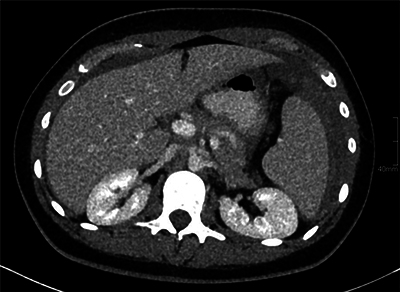
Contrast-enhanced abdominal CT shows a haemoperitoneum. A hyperdense, perisplenic collection with layering suggests an acute perisplenic haematoma.

**Figure 3:**
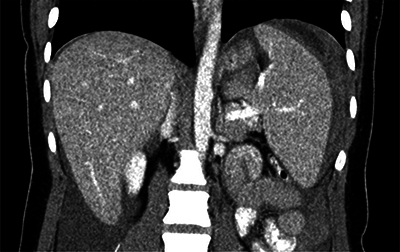
Contrast-enhanced abdominal CT shows a haemoperitoneum and splenomegaly. A hyperdense, perisplenic suggests an acute perisplenic haematoma.

A contrast-enhanced computed tomography (CT) of the abdomen revealed a haemoperitoneum. A hyperdense, layered perisplenic collection with a density of 60 HU was suggestive of an acute perisplenic haematoma. There were no signs of active bleeding. There was mild splenomegaly (Figures 2 and 3).

The patient underwent an urgent abdominal laparoscopic exploration. A four-quadrant haemoperitoneum and a large perisplenic haematoma (with multiple blood clots) were confirmed. During surgery, two litres of blood were aspirated, and the perisplenic blood clots were not manipulated. The surgeons didn’t perform a splenectomy. No active bleeding was visualised.

Post-operative therapy consisted of optimising the haemodynamic situation. The patient left the intensive care unit (ICU) after 5 days and fully recovered.

## Comments

Infectious mononucleosis is a contagious illness induced by the EBV. It infects B-cells in the lymphoid tissue and is spread via saliva. Infected people are usually asymptomatic, others present with fever, pharyngitis, lymphadenopathy, malaise and fatigue. Normally, it is a self-limiting condition [1].

Complications such as myocarditis, pericarditis, pancreatitis and splenic rupture are rare. Splenic rupture occurs in 0.1% to 0.5% of patients. Infectious mononucleosis causes spleen enlargement and intrinsic changes in the splenic parenchyma, resulting in greater susceptibility to rupture [1].

Splenic rupture in EBV-infected patients is mostly atraumatic. They mostly present with an acute onset of abdominal pain located in the left upper quadrant. Presentation with pleuritic left-sided chest pain or left shoulder pain is less common.

Lab results in infectious mononucleosis include lymphocytosis and elevated liver function tests. The monospot test is a very specific antibody test to diagnose EBV infection and is used as the golden standard [1].

Common radiologic findings in an EBV-associated atraumatic splenic rupture are haemoperitoneum, perisplenic haematoma and splenomegaly.

In case of haemodynamic stability, treatment is conservative with fluid resuscitation and analgesia. In case of haemodynamic instability, explorative abdominal surgery is recommended, and a splenectomy can be required [1].

This case highlights the importance of diagnosing EBV-associated (atraumatic) splenic rupture to prevent a potentially fatal outcome.
